# Mastoiditis and Gradenigo’s Syndrome with anaerobic bacteria

**DOI:** 10.1186/1472-6815-12-10

**Published:** 2012-09-14

**Authors:** Chris Ladefoged Jacobsen, Mikkel Attermann Bruhn, Yousef Yavarian, Michael L Gaihede

**Affiliations:** 1 Department of Otolaryngology, Head and Neck Surgery, Aalborg Hospital - Aarhus University Hospital, Aalborg, Denmark; 2 Department of Radiology, Aalborg Hospital - Aarhus University Hospital, Aalborg, Denmark

**Keywords:** Gradenigo’s syndrome, Acute mastoiditis, Apical petrositis, Acute otitis media, Abducens palsy, Fusobacterium necrophorum

## Abstract

**Background:**

Gradenigo’s syndrome is a rare disease, which is characterized by the triad of the following conditions: suppurative otitis media, pain in the distribution of the first and the second division of trigeminal nerve, and abducens nerve palsy. The full triad may often not be present, but can develop if the condition is not treated correctly.

**Case presentation:**

We report a case of a 3-year-old girl, who presented with fever and left-sided acute otitis media. She developed acute mastoiditis, which was initially treated by intravenous antibiotics, ventilation tube insertion and cortical mastoidectomy. After 6 days the clinical picture was complicated by development of left-sided abducens palsy. MRI-scanning showed osteomyelitis within the petro-mastoid complex, and a hyper intense signal of the adjacent meninges. Microbiological investigations showed Staphylococcus aureus and Fusobacterium necrophorum. She was treated successfully with intravenous broad-spectrum antibiotic therapy with anaerobic coverage. After 8 weeks of follow-up there was no sign of recurrent infection or abducens palsy.

**Conclusion:**

Gradenigo’s syndrome is a rare, but life-threatening complication to middle ear infection. It is most commonly caused by aerobic microorganisms, but anaerobic microorganisms may also be found why anaerobic coverage should be considered when determining the antibiotic treatment.

## Background

Gradenigo’s Syndrome (GS) is a clinical triad of the following conditions; otitis media, pain in the distribution of the first and second division of the trigeminal nerve and ipsilateral abducens palsy. It was originally described in 1907 by Guiseppe Gradenigo [1]. Before the antibiotic era it was not uncommonly seen as a complication to acute otitis media (AOM) and mastoiditis. The symptoms occur as the infection spreads to the petrous apex of the temporal bone, where the sixth cranial nerve and the trigeminal ganglion are in close proximity only separated by the dura mater. The involvement of the sixth cranial nerve is seen as a reaction caused by the adjacent inflammation, as the nerve passes through Dorello´s canal under the petroclinoid ligament [2].

The full triad of symptoms in GS may not always be present. For instance, the absence of abducens palsy does not rule out apical petrositis. Radiologic evaluation by computed tomography (CT) and magnetic resonance imaging (MRI) are helpful tools in the diagnosis and management of GS, as well as they may exclude differential diagnoses like septic sinus thrombosis or other non-infectious entities [3,4]. With the widespread use of antibiotics the incidence of apical petrositis is now rare, reportedly two per 100,000 children with acute otitis media [5].

We report a case of AOM complicated by mastoiditis and apical petrositis presenting as Gradenigo’s syndrome.

## Case presentation

A 3-year-old healthy girl with no prior medical history was admitted to the pediatric department after 4 days with a high fever (39–40.4°C; 102.9-104°F) and left-sided otorrhea. Upon admission the child was in poor condition, dehydrated, pyretic and pale. Physical examination showed left mastoid tenderness with retroauricular erythema, edema and fluctuation. Furthermore, examination of the eyes revealed normal movements and reflexes; there were no meningeal signs, changes in consciousness or other neurological findings. No cervical lymph nodes were enlarged. Initial blood samples showed a C-reactive protein of 256 mg/L and intravenous antibiotic treatment with benzyl penicillin was initiated.

The child was transferred to our ORL department, where otomicroscopy showed edema of the external auditory canal and a bulging, hyperemic tympanic membrane. These findings led to surgical drainage of the abscess under general anesthesia and insertion of a ventilation tube into the left tympanic membrane. Mucopurulent material from the abscess and the middle ear was sent for microbiological examination. The day after the operation the child showed signs of improvement with remission of fever as well as the retroauricular edema and erythema, and the anorexia diminished. However, later the same day, fever relapsed (39.0°C; 102,2°F), as well as progression of the erythema and swelling around the incision. Because of the rapid deterioration acute mastoidectomy and drainage were performed.

**Figure 1:**
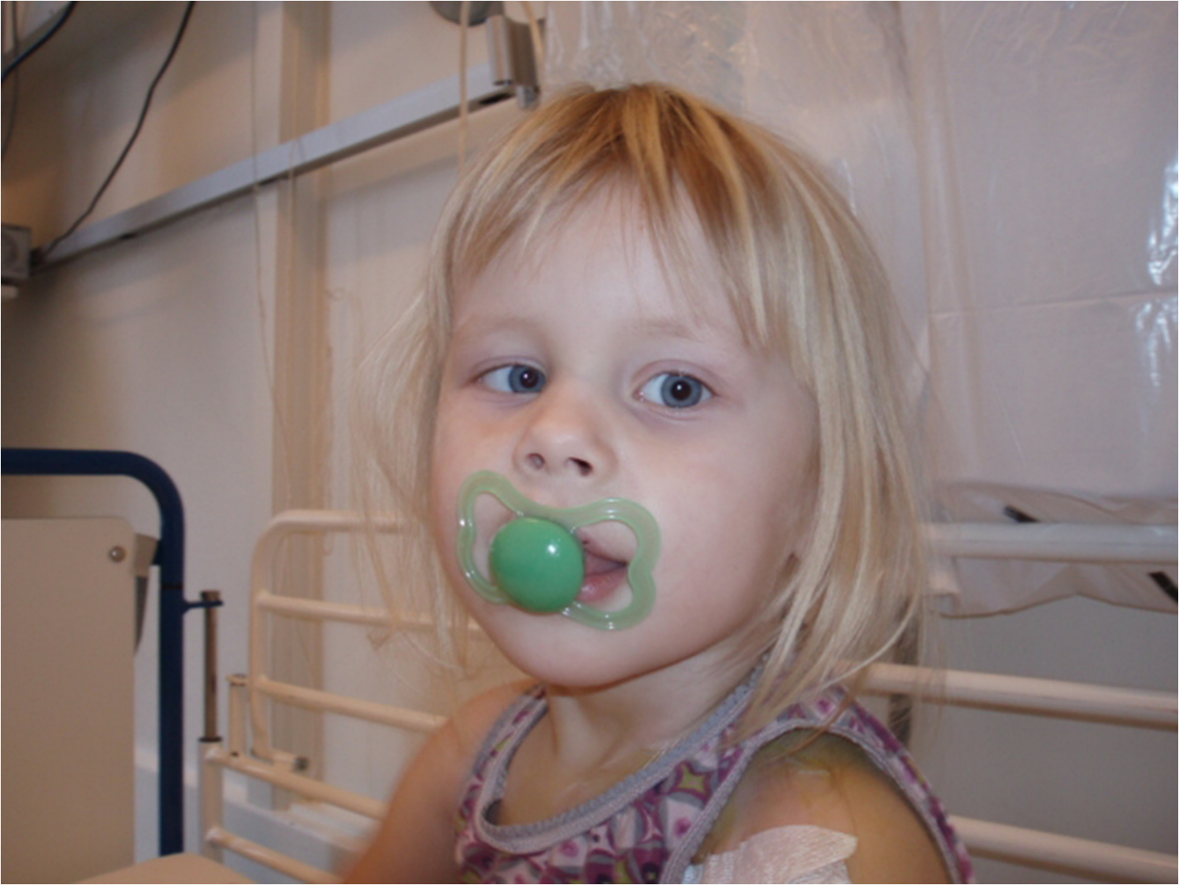
Left-sided abducens palsy at its onset 6 days after mastoidectomy.

During the following days continuous clinical improvement was registered. Six days after the operation the child had problems maintaining balance, and the parents noticed that she had developed a slight strabismus. No headache or involvement of the trigeminal nerve were found. Physical examination showed normal visus on both eyes, but discrete left-sided papillar edema and abducens palsy (Figure 1).

**Figure 2:**
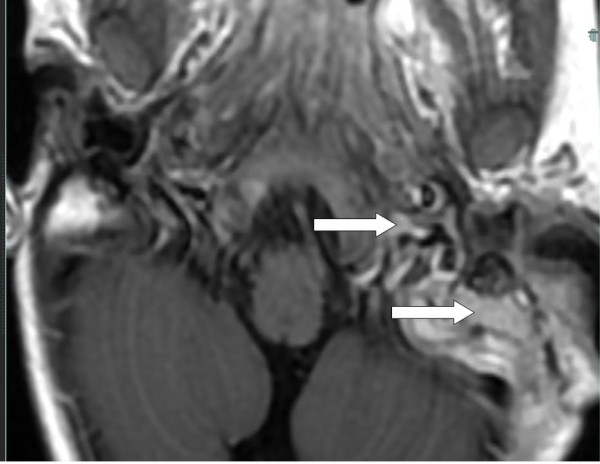
MRI horizontal section T1 + Gadolinium. Arrows show areas with enhancement in left pars petrosa.

**Figure 3:**
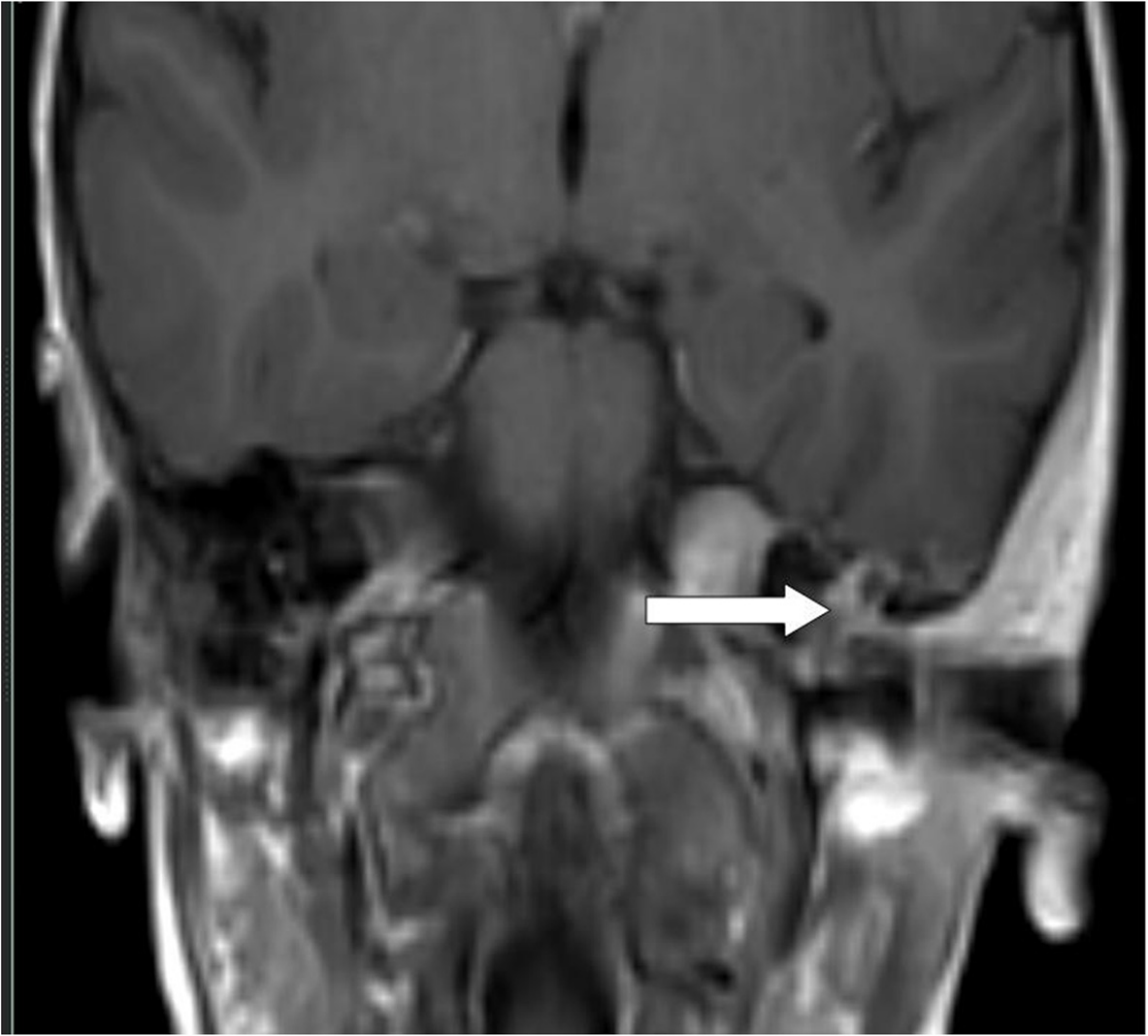
MRI coronal section T1 + Gadolinium. Arrow shows area with enhancement in left pars petrosa.

**Figure 4:**
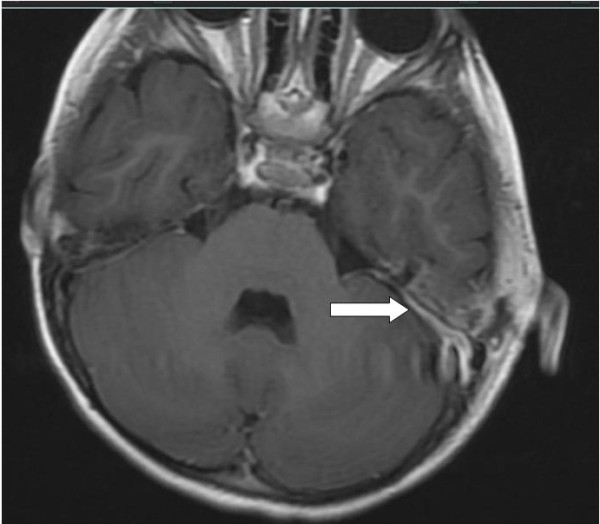
MRI horizontal section T1 + Gadolinium. Arrow shows local thickening and enhancement of the dura.

In order to exclude the possibility of sinus thrombosis, an MRI scanning was performed. This demonstrated osteomyelitis within the petro-mastoid complex (Figures 2 and 3); further, thickening and enhancement of the adjacent meninges were demonstrated (Figure 4), whereas there were no signs of sinus thrombosis. Finally, no intracranial abscesses were found after contrast injection.

The pus culture isolated from the mastoid abscess revealed growth of Staphylococcus aureus sensitive for dicloxacillin and cefuroxime, but resistant to penicillin and Fusobacterium necrophorum was demonstrated sensitive to metronidazole and penicillin. The child was discharged after a total of 20 days of intravenous antibiotics as a combination of cefuroxime and metronidazole. By the time of 8 weeks clinical follow-up examination there were no signs of recurrent infection or abducens palsy.

## Conclusions

GS as a result of petrositis is rarely seen after the introduction and widespread use of antibiotics. It remains, however, a serious and potentially fatal complication to AOM and acute mastoiditis.

Whereas the pneumatisation of the mastoid cells of the temporal bone is almost universal, the pneumatisation of the petrous apex varies and is only found in one third of adult patients; in such cases it may provide a path for AOM to spread also medially causing apical petrositis [4]. In addition, this condition may also be a result of direct extension of the infection through bony destruction or hematologically through venous channels causing true osteomyelitis in the non-pneumatized areas of the petrous bone [6]. Due to the central location of the petrous apex, apical petrositis may rapidly develop into severe and life threatening complications like meningitis, brain abscess, lateral sinus thrombosis, empyema and cranial nerve palsies [4-6]. The delay between otologic symptoms and cranial nerve involvement varies from 1 week to 2–3 months [7]. In our case, the time between the onset of the initial symptoms and the registration of the abducens palsy was two weeks.

We found the causative pathogens to be Staphylococcus aureus in combination with Fusobacterium necroforum found in both the middle ear and the mastoid cavity. Staphylococcus aureus are frequently found in acute mastoiditis (8.6 %), only surpassed by Pseudomonas aeruginosa (11.8 %), Streptococcus pneumoniae (9.9 %) and Streptococcus pyogenes (9.2 %) [8]. It has been argued that S. aureus has a greater tendency to invade bone, since it has been found in more cases with osteomyolitis [8]. The demonstration of Fusobacterium necrophorum in GS is more unusual, and to our knowledge this has only been reported in two cases previously [9,10]. Fusobacterium necrophorum is an anaerobic, non-motile Gram-negative rod, usually found in the oral flora, the gastro-intestinal tract as well as the genito-urinary tract in females. It usually does not invade mucosal surfaces in healthy individuals, but if the host´s defence system is compromised it has been known to cause a variety of rapidly progressing serious infections including bacteremia; these clinical conditions are known as necrobacillosis [11].

The demonstration of Fusobacterium necrophorum is difficult, since the cultivation is complex and depends on a prolonged incubation period; this may cause an underestimation of its clinical demonstration [12] however the cultivation of anaerobic microorganism should be considered. Thus, when empiric antibiotical treatment is started, it can be recommended to include both a potent anti-staphylococcal agent as well as metronidazole to cover anaerobic organisms.

CT scan is the first choice of imaging, since it is widely available and has a high sensitivity for detection of changes in bone structures including lesions in the petrous apex where GS in most cases will appear [4]. Furthermore it may detect the presence of intercranial abscesses, though it is less sensitive than MRI. An MRI is useful in evaluating the extent of the lesion of the petrous apex localised on the CT scan, as well as demonstrating meningeal involvement. Moreover, MRI is superior in detecting intracranial complications [3,4,13]. An MRI angiography may be performed to rule out signs of sinus thrombosis.

GS is a rare, but life-threatening complication to middle ear infection that should be taken into consideration when atypical symptoms develop after AOM. Radiological modalities such as CT and MRI should not be delayed and CT should be regarded as first choice of imaging when GS is suspected. Treatment should include drainage of the middle ear and mastoidectomy as well as intravenous broad spectrum antibiotics. GS is most commonly caused by aerobic microorganisms, but it may also be found in interaction with anaerobic microorganisms. Due to the severity of related complications we suggest that antibiotic treatments include anaerobic coverage.

## Consent

Written consent, for publication of the clinical details and clinical images was obtained from the parent of the patient. A copy of the consent form is available for review by the Editor of this journal.

## Abbreviations

GS: Gradenigo’s Syndrome; AOM: Acute otitis media; CT: Computed tomography; MRI: Magnetic resonance imaging.

## Competing interests

The authors declare that they have no competing interests.

## Authors’ contributions

CJ carried out the writing of the manuscript, acquisition of informed consent and literature research and approval of all images in the case report. MB helped in outlining and modification of the manuscript as well as selection and acquisition of photographs used in the case report. YY carried out the radiological evaluation, selection and description of the MRI images and modification and approval of the imaging section. MG supervised, commented and helped in the revision and final approval of the manuscript. All authors have read and approved the final manuscript.

## Financial and non-financial disclosure

None.

## Pre-publication history

The pre-publication history for this paper can be accessed here:

http://www.biomedcentral.com/1472-6815/12/10/prepub
